# *Punica granatum* as Anticandidal and Anti-HIV Agent: An HIV Oral Cavity Potential Drug

**DOI:** 10.3390/plants11192622

**Published:** 2022-10-05

**Authors:** Maira Huerta-Reyes, Luis A. Gaitán-Cepeda, Luis O. Sánchez-Vargas

**Affiliations:** 1 Unidad de Investigación Médica en Enfermedades Nefrológicas, Hospital de Especialidades “Dr. Bernardo Sepúlveda Gutiérrez”, Centro Médico Nacional Siglo XXI, Instituto Mexicano del Seguro Social, Ciudad de México 06720, Mexico; 2 Departamento de Medicina y Patología Oral y Maxilofacial, División de Estudios de Postgrado e Investigación, Facultad de Odontología, Universidad Nacional Autónoma de México, Ciudad de México 04510, Mexico; 3 Laboratorio de Bioquímica y Microbiología Oral, Facultad de Estomatología, Universidad Autónoma de San Luis Potosí, San Luis Potosí 78290, Mexico

**Keywords:** *Punica granatum*, pomegranate, HIV, *Candida*, oral cavity, punicalagin, polyphenols, anthocyanins, gallotannins

## Abstract

The oral cavity is crucial from diagnosis to adherence to HAART therapy in the HIV/AIDS population; consequently, drugs that can maintain healthy conditions in the oral cavity are necessary for patients with HIV/AIDS. *Punica granatum* (pomegranate) is a tree that has been employed extensively for centuries in the traditional medicine of ancient cultures for the treatment of a wide range of diseases, including oral and dental diseases. In recent decades, its potent anticandidal properties have been shown, especially on *Candida albicans*, the cause of the most common clinical manifestation in HIV patients. The present work contributes to the review of the anti-HIV and anticandidal properties of the plant species *P. granatum* as involved with the oral cavity. The literature reviewed revealed that crude extracts of pomegranate and its main isolated compounds possess inhibitory activity on different HIV targets, including binding viral proteins and the three replicative HIV enzymes. In addition, in the literature reviewed, pomegranate exhibited anticandidal effects on 10 different species. Thus, pomegranate appears to be an excellent candidate to explore and incorporate into the treatment of the oral cavity of HIV/AIDS patients, in that, in addition to its pharmacological effects such as antiviral and anticandidal, pomegranate represents an easily available, inexpensive, and safe natural source.

## 1. Introduction

Oral–dental diseases comprise a major public health problem due to their high prevalence and incidence throughout the world, where the unprivileged population bears the greatest burden. It has been calculated that over 3.5 billion people experience oral diseases through childhood to adolescence, adulthood, and until old age [1,2]. Close to 90% of the world’s population experiences dental and/or oral disease during their lifetime. Dental and oral mucosa diseases significantly impact socioeconomic aspects in terms of healthcare costs, academic time, and work time [3]. In this manner, the role of oral health in the everyday life of people is crucial for general health and well-being [1], due to the profound effect of pain, problems with eating, chewing, smiling, and missing or damaged teeth. Moreover, poor oral health has been related to chronic diseases such as cancer and diabetes, and particularly to HIV/AIDS [4,5].

*Candida* species form part of the normal oral microbiota, however, under conditions of immunosuppression, and especially in HIV/AIDS, an exponential increase in colonization causes oral candidiasis (OC). OC is the most common clinical manifestation in HIV patients, furthering the AIDS transition in both patients treated with Highly Active AntiRetroviral Therapy (HAART) and in untreated patients [6,7]. Thus, candidiasis is considered an important marker of immune suppression (CD4^+^ counts < 200/mm^3^) and also a predictor of progression to AIDS [8,9]. OC also implies discomfort during eating, limitation in chewing, and consequently a reduction in food intake. Thus, malnutrition can be developed, which is a relevant mortality risk factor in patients with HIV, especially children, because of the weakened immune state [6].

*C. albicans* is the most common species isolated in oral mucosa, while in oropharyngeal candidiasis, it is *C. glabrata* followed by *C. dubliniensis*. *C. albicans* and *C. glabrata* are known for their development of resistance to azole antifungals; therefore, the management of OC remains difficult in HIV/AIDS [8]. While a number of antifungal agents are available for the treatment of candidiasis, Fluconazole continues to be the first-line therapy. In consequence, the emergence of antifungal resistance in patients with continual OC or long-term use of antifungals demands alternative and effective antifungal agents [10].

Particularly in HIV/AIDS, the oral cavity represents a baseline in the diagnosis of the disease, in which, as we previously mentioned, candidiasis is the most important infection; however, another aspect of HIV-related illness comprises the ulcerative and periodontal disorders that are involved in the staging of HIV infection [11]. Furthermore, it is noteworthy that severely compromised oral health in patients with HIV/AIDS showed difficulty in chewing, swallowing, in maintenance of the salivary flow, and in tasting foods. All of these aspects are not only involved in general health and well-being, but also impact negatively the adherence to HAART therapy, since the majority of schemes for HIV treatment involve the oral administration of drugs [11,12]. Therefore, oral health in HIV/AIDS patients remains fundamental and an integral hallmark of the disease, and drugs that can maintain healthy conditions in the oral cavity are necessary for the HIV/AIDS population.

Despite that HAART therapy has improved the length and quality of life for patients with HIV/AIDS through the reduction in the number of cases of AIDS progression and a diminution in AIDS-related morbidity and mortality, positioning it as a chronic manageable disease [13], recent reports indicate that exposure to HAART therapy may have adverse effects, independent of the HIV stage [14]. These adverse effects cause diseases such as neurological (peripheral neuropathy, dementia), hematological (anemia), metabolic (hyperlipidemia, lipodystrophy), gastrointestinal (gastritis, anorexia, diarrhea), hepatic, and cutaneous [14,15]. Therefore, the population living with HIV/AIDS requires lifelong treatment with sustained potency, limited or absent toxicity, and reduced cost [15], but also drugs that can exhibit more than one mechanism of action and facilitate adherence to the antiretroviral (ARV) treatment. In this regard, natural products have been providing chemical identities with promising anti-HIV properties for the development of novel drugs during the last decades. Natural-compound-based anti-HIV therapies have become more efficient than HAART, with notoriously fewer or the absence of secondary effects [16] and, in addition, with more than one site of action. Even more so, a dual effect such an anti-HIV and anticandidal would be highly valuable, in that it would also impact adherence to pharmacological treatment.

Among the most studied natural products over recent decades, we find the prominence of the plant species *Punica granatum*, known as pomegranate, an ancient, mystical, unique fruit originally from Asia but currently cultivated around the world. Pomegranate has been recognized as a “miracle fruit” because its seeds are consumed as food and juice, and also as a functional food where the juice constitutes 36–63% while the edible part amounts to 57–85% of the entire fruit [17,18]. The whole fruit possesses abundant amounts of natural antioxidants that provide a number of health benefits, and Ayurveda considers the pomegranate as a “pharmacy unto itself”. In traditional medicine, not only the fruit, but also the whole plant of pomegranate, has been employed in the treatment of a variety of diseases, the most common of these being diarrhea, intestinal worms, ulcers, and dental diseases [17,19]. Nevertheless, recent investigations reported a great variety and number of pharmacological properties of pomegranate for the treatment of diabetes, cancer, hypertension, hyperlipidemia, atherosclerosis, and oral diseases, without adverse effects [19]. A number of phytochemicals have been identified as bioactive in pomegranate, from which polyphenols such as anthocyanins and other flavonoids as well as gallotannins are those most commonly reported [20]. Thus, pomegranate represents a potential source of bioactive phytochemicals that may become the base of new drugs. For these reasons, the present manuscript contributes necessary information for the research of bioactive molecules extracted from pomegranate that are useful in the treatment of oral cavity diseases, and particularly, of the oral cavity in patients with HIV/AIDS. This review offers a general overview of up-to-date knowledge of the chemical composition and anti-HIV and anticandidal properties of the plant species *P. granatum* involved in the oral cavity in order to collaborate in the development of novel anti-HIV agents that will be useful in future clinical practice.

## 2. Brief Overview of HIV and *Candida* spp.

Worldwide, more than 38 million people live with HIV. This fact reveals that the HIV-infection pandemic is far from being controlled and eventually eradicated. Therefore, developing a cure for HIV remains a major global health priority. Globally, HIV infection represents a significant health burden, in which the current anti-HIV treatments are not curative, even though their principal goal is to suppress viral replication until reaching undetectable levels. Other treatment targets block the viral–lymphocyte fusion [21]. The target of ART drugs is the structural and enzymatic components of both the virus and the CD4 lymphocyte cells. The first anti-HIV-approved drug was Zidovudine in 1987. From that moment to date, over 39 antiretroviral drugs have been classified based on their pharmacological action and resistance outline in six different categories, as follows: (1) nucleoside analogue reverse transcriptase inhibitors; (2) non-nucleoside reverse transcriptase inhibitors; (3) integrase strand transferase inhibitors; (4) protease inhibitors; (5) fusion inhibitors; and (6) co-receptor antagonists [22]. For example, the three replicative HIV enzymes, that is, reverse transcriptase (RT), integrase (IN), and protease (PR), are druggable targets; moreover, RT and IN have been suggested for dual novel inhibitors. Some new approaches such as Designed Multifunctional Ligand (DML) recruits several targets that are the site of action of only one substance or compound. One of the most notorious advantages of these multifunctional ligands as antiretrovirals is based on the viral replication cycle, which can be affected in two or more phases [23]. HAART, a multi-target drug scheme, has proven effective in treatment for AIDS. However, despite their efficacy and efficiency, ART drugs face challenges, e.g., toxicity, the development of drug-resistant HIV-1 strains, the expensiveness of the drugs, and the failure to eliminate the provirus in infected cells. Consequently, to achieve pharmacological success, many factors are involved, including the following: correct patient adherence to antiretroviral (ART) treatment; the availability of antiretroviral drugs; distribution of antiretrovirals; the development of HIV-resistant genotypes, among others. Thus, novel anti-HIV-1 drugs that can be effective in treating acquired immunodeficiency syndrome (AIDS) progression are needed [24]. In this case, the metabolites of certain plants could be a possible alternative.

The oral lesions related to HIV have been widely recognized since the start of the epidemic, and they are considered early clinical signs that may predict the disease progression of patients with AIDS. More than one-third of people living with HIV worldwide present oral manifestations. In developing countries, where the majority of HIV-positive patients reside, oral lesions, specifically oral candidiasis, acquire greater significance. Oral candidiasis is the most frequently found oral manifestation of HIV in several world regions, including in patients with HIV/AIDS undergoing HAART [25]. The most frequent oral mucosa disease in people living with AIDS (PLWA) is oral candidiasis, specifically the pseudomembranous clinical variety [26]. Therefore, *Candida* spp. arises as a highly potential pathogenic fungus in patients with HIV and bronchopulmonary diseases [27].

## 3. Generalities of *P. granatum*

*P. granatum* L. (Lythraceae) is known as pomegranate, grenadier, granada, granado, mangrano, dadima, granatapfelbaum, granatapfelstrauch, granatbaum, mogranj, romazeira, delima, and melograno, among others. *P. granatum* is a shrub or small tree 5–10 m in height, with leaves that are simple, bright green, and glabrous, ovate, or oblong. The flowers are funnel-shaped with 5–7 petals of an orange-red color. The most conspicuous characteristic of pomegranate is the fruit, which is rounded (berries) and is composed of two parts: an external hard pericarp and an inner mesocarp of a spongy nature. The inner mesocarp possesses numerous chambers that contain fleshy seeds [28,29]. *P*. *granatum* is one of only two species that belong to the genus *Punica* spp. The other species, *P*. *protopunica* Balf, is known as Socotran pomegranate and is an endemic plant of the Socotra Archipelago in the Indian Ocean, and that, according to recent studies, is considered to be the ancestor of *pomegranate* [30]. The origin of the pomegranate was probably Iran and the Northwestern Himalayas, but at present, it is cultivated throughout India, the Mediterranean region, Northern Africa, Central Asia, and in North America, principally in the states of Arizona and California, and in Mexico.

Currently, India is the largest producer of pomegranate (2.7 million metric tons), followed by China (0.3 million metric tons) and Turkey (0.22 million metric tons) [31]. Worldwide, more than 500 varieties or cultivars of pomegranate have been recognized, and among these, only 50 are commonly grown. These cultivars are the result of human selection based on market demands that include taste, juice content, resistance to plagues, and tolerance to climatic conditions, among others [32]. Thus, at present, new cultivars of pomegranate are being developing worldwide, but especially in Latin American countries [33].

*P. granatum* L. is a plant with a powerful history and has been noteworthy in many religions worldwide; for example, it is said to have flourished in the Garden of Eden, and was a symbol of sanctity, fertility, good luck, and abundance, as it is referred in the Bible. In Greek mythology, pomegranate was the symbol of life, regeneration, and marriage. In Ancient Egypt, the pomegranate represented prosperity and ambition. In Buddhism, the pomegranate is considered one of the three sacred fruits of the essence of beneficial influences. Its impact has also been observed as the symbol of the Roman Emperor, Maximilian, and in the name of the Spanish province denominated Granada [28,34,35].

Since ancient times, the medicinal uses of pomegranate have been recorded. All organs of pomegranate exhibit medicinal properties, including its fruits, flowers, leaves, and roots. The variety of medicinal uses of pomegranate is extensive, but perhaps the most reported traditional use of pomegranate worldwide has been as a vermifuge, or for removing intestinal parasites through the ingestion of fresh or dried root bark preparations [36,37]. Due the potent astringent properties of the decoctions prepared with different parts of the fruit, such as pomegranate peel or arils, this is considered by Lansky et al. [36] as the second most common traditional use of pomegranate. These decoctions are traditionally employed to cure diarrhea or dysentery, and interestingly, the decoction can be drunk or used as a mouthwash, as a douche, or as an enema [36,38]. Other traditional uses of pomegranate are of a broad variety, as follows: in the treatment of dental diseases, ulcers, and leprosy; as a remedy for cut wounds, bronchitis, and digestive problems; as a male sexual power regenerator; to prevent fertility in women; to treat diabetes [39]; in the treatment of abdominal pain; to stop grey hair in young people; to treat loss of appetite, indigestion, snakebite, kidney diseases, and hemorrhoids [18,36].

### Phytochemical and Pharmacological Profile of P. granatum

Pomegranate plants are a rich source of chemical entities used as medicine from time immemorial. Any part of the plant may contain active components that have been described in their majority as secondary metabolites [40]. The role of secondary metabolites in plants is recognized as fundamental in the following: in the adaptation process to changing environmental conditions; in the defense against herbivores and microorganism attacks; in competition with other plant species; and also, in reproductive processes such those that attract pollinators and seed dispersers. Consequently, the synthesis and accumulation of secondary metabolites in plants is determined by several environmental conditions, such as temperature, light, soil composition, salinity, altitude, and water, as well as seasonality, among others [41,42]. Hence, the presence and quantity of the active constituents could be altered by changes in environmental conditions and by the cultivation type, affecting the medicinal properties of the plants. Thus, a number of studies that describe the general phytochemical content of the pomegranate from different regions worldwide have been performed during the last decades, and these primary chemicals of pomegranate are included in Table 1. Nonetheless, some reports mention that the tannin (punicalagin) and anthocyanin content of pomegranate, especially delphinidin, cyanidin, and pelargonidin, is crucial for maintaining some of the medicinal properties of the pomegranate fruit [35,41].

As can be noted in Table 1, the chemical composition of *P. granatum* is extensive and diverse, and a number of pharmacological activities that have been attributed to these chemicals have been reviewed elsewhere. Nonetheless, the most predominant phytochemicals recognized in pomegranate are gallotannins and anthocyanins [43]. Among these polyphenolics, some compounds, such as punicalagin and ellagic acid, are particularly interesting, because they are reported to be bioactive in the antioxidant, anti-inflammatory, anticancer, and antimicrobial activities exhibited by *P*. *granatum*, which are also the main pharmacological properties studied for the species [63]. Moreover, punicalagin has been identified as the major active component of pomegranate extracts [64], and with a variety of biological activities of its own, as we show in Table 2.

Nevertheless, not only antioxidant, anti-inflammatory, anticancer, and antimicrobial activities have been reported for extracts of *P. granatum*, but also, due to studies in recent decades, antihypertensive, anti-atherosclerosis, antiviral, anti-mutagenicity, antinociceptive, and anti-Alzheimer activities have been described [18,28,43,65]. Other biological activities in which pomegranate is active include lipid metabolism regulation, skin protection, glucose-metabolism regulation (diabetes), osteoporosis, obesity, erectile dysfunction, gastroprotection, hepatoprotection, and dental health [18,34,66].

**Table 2:** Principal pharmacological properties of punicalagin, the major active compound in *P. granatum*.

Pharmacological Effect	Assay	Reference
Antioxidant	Exhibited an IC_50_ = 1.9 ± 0.2 μg/mL in DPPH radical scavenger test. It also exerted significant ferrous chelating activity, lipid peroxidation inhibition, ferric-reducing antioxidant power, scavenging of superoxide anion, and reducing power ability in a dose-dependent manner.	[67,68,69]
Anti-inflammatory	Decreased pro-inflammatory cytokines (TNF-α, IL-1β, and IL-6) and down-regulated iNOS and COX-2 expression. Played a regulatory role in NF-κB, MAPK, IL-6/JAK/STAT3, and PI3K/Akt/mTOR signaling pathways.	[64,70]
Anticancer	Apoptotic and cytotoxic effects were shown by nano-prototypes on MCF7 and MDA-MB-231 breast cancer cell lines. Expression levels of Cas-3 and Bax were up-regulated, while the expression levels of BCL-2, NF-ĸB, and PI3k were down-regulated.	[71]
	Exhibited cytotoxic effects by suppressing proliferation and promoting apoptosis and autophagy through the activation of caspase cascade. Bax and Bcl-2 were altered and via mTOR/ULK1 exhibited regulation of autophagy in acute leukemia.	[72]
	Toxic effects and reactive oxygen species generation were induced in lung cancer A549 cells. Alterations in the mitochondrion membrane potential and apoptotic morphological changes were recorded. Punicalagin also inhibited STAT-3 translocation and induced apoptosis by inhibiting expression of Bcl-2 and enhanced expression of Bax, cytochrome-c, caspase-9, and caspase-3 in A549 cells.	[73]
	Possessed chemopreventive and chemotherapeutic effects against human ovarian cancer through the inhibition of β-catenin signaling pathway. It also inhibited the cell viability of A2780 ovarian cells in a dose-dependent manner, as well as induced apoptosis by the up-regulation of Bax and down-regulation of BCL-2.	[74]
	Inhibited the viability of cervical cancer cells in a dose-dependent manner via stimulating mitochondrial-mediated apoptosis. Stimulated cell apoptosis by suppressing NF-kappa B (NF-kB) activity, which was also affected by punicalagin, blocking cancer cell progression.	[75]
Antimicrobial	MIC values between 0.3 and 1.2 µg.mL^−1^, where the spectrum of activity targeted Gram-positive and Gram-negative bacteria, as well as a yeast strain. The three Gram-positive strains were *Pseudomonas aeruginosa* ATCC 9027, *Salmonella enteritidis* LC 216, and *Escherichia coli* ATCC 25922. The 13 Gram-positive bacteria strains comprised the following: *Staphylococcus epidermidis* ATCC 12228; *Staphylococcus xylosus* LC 57; *Staphylococcus aureus* ATCC 6538; *Bacillus cereus* CIP; *Lactobacillus sakei* ssp. *sakei* ATCC 15521; *Lactobacillus plantarum* CECT 4185; *Pediococcus acidilactici* LC P1; *Enterococcus faecium* DSMZ 10,663; *Enterococcus faecalis* ATCC 19,433; *Enterococcus mundtii* LC E23; *Enterococcus sulfureus* LC E28; *Enterococcus casseliflavus* LC E1; and *Enterococcus columbae* LC E2. The yeast strain employed was *Candida albicans* ATCC 10231.	[76]
	Induction of an increase in the extracellular concentrations of potassium and a release of cell constituents, but also an increase in intracellular pH, cell membrane depolarization, and damage in the structure of the cell membrane of *Salmonella* Typhimuriume.	[77]
	Exerted activity against *Plasmodium falciparum* D6 and W2 clones with IC_50_ values of 7.5 and 8.8 μM. Revealed antimicrobial activity against *Escherichia coli*, *Pseudomonas aeruginosa*, *Candida albicans*, *Cryptococcus neoformans*, Methicillin-resistant *Staphylococcus aureus* (MRSA), *Aspergillus fumigatus*, *Mycobacterium intracellulare*, *P. aeruginosa*, and *C. neoformans*.	[78]
Antiviral	Inhibited replication of influenza A and B viruses (several strains), including Oseltamivir-resistant virus (NA/H274Y) with a low micromolar IC_50_ value in tissue culture.	[79]
	Inhibitory action against the SARS-CoV-2 3CL-protease in a dose-dependent manner, with IC_50_ = 6.192 μg/mL for punicalagin. When punicalagin was combined with zinc sulfate monohydrate (punicalagin/Zn-II), the inhibitory activity became extremely strong in 3CL-protease activity (more than 4-fold approx.) No toxicity was observed by punicalagin, Zn-II, or punicalagin/Zn-II.	[80]
	Reduced the viral cytopathic effect of enterovirus 71 on rhabdomyosarcoma cells with an IC_50_ = 15 μg/mL. In mouse treatment with a lethal dose of enterovirus 71, punicalagin reduced mortality and relieved clinical symptoms by inhibiting viral replication.	[81]

## 4. *P. granatum* Products That Are Relevant for Oral Diseases

In recent years, an increasing number of reports have recognized that oral health exerts an important effect on the general quality of life. Some chronic and systemic diseases are related to poor oral health, which often worsens these diseases [82]. The microorganisms detected in the oral cavity include bacteria, virus, fungi, and protozoa, among which many species of bacteria are innocuous. However, a few of the most common oral diseases that affect humans are caused by these microorganisms, that is, dental caries and periodontitis. In consequence, oral hygiene has relevance in the prevention and treatment of oral infections through the daily use of chemical agents in toothpastes and mouth rinses [83]. Nevertheless, oral microbes have developed a considerable increase in clinical resistance to drugs such as antibiotics and antifungals, which have been advantageous through the indiscriminate use of antibiotics and azole drugs. Thus, failure to respond in the current scenario of bacteria and fungus diseases calls for the search for novel antimicrobial and antifungal substances that also have no side effects for oral disease pathogens [84,85]. As we noted previously, *P. granatum* has been widely reported in traditional use for the treatment of dental diseases, and a number of studies concerning the effects on different oral diseases have been published in recent decades, during which a variety of mechanisms of action on bacteria, viruses, and fungi were found. However, *P. granatum* is different from other plants, not only due its effectiveness and lack of side effects, but also because all parts of the plant can be used for pharmacological effects in oral diseases [86]. Thus, in Table 3, we present some of the most representative activities described for *P. granatum* in oral diseases, in which the fruit is the sole utilized part of the plant for these diseases.

## 5. Anti-HIV and Anticandidal Properties of *P. granatum*

In the current HAART therapy utilized for the treatment of HIV, the initial schemes include a combination of three or more ARV drugs from at least two different HIV drug classes that target different structures and steps of the HIV life cycle [12]. The goal of combination ARV is to contain the HIV viral load in plasma under the limit of detection, and also to re-establish the immune function, mainly by elevation in the number of CD4+ T cells [108]. Thus, bioactives that possess more than one target of action or their effects involve the decrease or inhibition of viral replication or the restoration of the immune system, an attractive result because of their potential as anti-HIV drugs. Therefore, *P. granatum* emerges as an interesting candidate for the future development of anti-HIV drugs due its anti-HIV and anticandidal properties reported in recent years [109].

In this manner, the anti-HIV activities of pomegranate have been reported for all organs of the plant and in different steps of the viral cycle of replication. The juice of the pomegranate exhibited inhibitory activity against HIV-1 in experiments that employed CD4+ T cells and CXCR4 as cell receptors. Pomegranate juice was able to block HIV-1 binding to CD4+ T and the CXCR4/CCR5 receptor. In the same investigation, one or more components of pomegranate juice bound strongly or irreversibly to the CD4+ T binding site on HIV-1 envelope glycoprotein gp120 [110]. The same authors also proposed that a topical microbicide applied vaginally (and possibly rectally) may be used for HIV prevention and that it could potentially be manufactured from pomegranate juice, in that HIV-1 entry inhibitors from pomegranate juice form a complex that successfully adsorbs onto a cornstarch base [111].

On the other hand, a screening of hydrolyzed peptides extracted from 111 Asian medicinal plants were tested against the HIV-1 RT. Only three species exhibited a potent anti-HIV-1 RT percentage of inhibition, and among these, in the third highest percentage, peptides extracted from the fruit peel of *P. granatum* showed 96.48% [112].

Another study evaluated the inhibition of the HIV-1 RT and HIV-1-IN enzymes of the ethanolic extract obtained from areas of the non-edible parts (leaves, bark, and fruit peels) of *P. granatum*. The IC_50_ values exhibited by all the pomegranate extracts revealed a strong inhibitory activity on HIV-1 RT and HIV-1 IN, with ranges of 0.22–0.85 µg/mL and 0.12–0.5 µg/mL, respectively. The bark extract exerted the highest inhibitory activity on HIV-1 RT, while the highest leaf extract inhibitory activity was exerted on HIV-1-IN. In this same investigation, the compounds detected in the pomegranate extracts were isolated and tested individually against the HIV-1 RT and HIV-1-IN enzymes. In the leaf extract, ellagic acid, flavones, and triterpenoids were identified, while hydrolyzable tannins, such as punicalagin, in addition to ellagic acid, were isolated from the bark and the peel extracts. Punicalagin and ellagic acid exhibited potent inhibition on both HIV-1 replicative enzymes: IC_50_ values ranged from 0.12–1.4 µM and from 0.065–0.09 µM for the RT and IN inhibition, respectively. In the case of luteolin and apigenin (flavonoids), inhibitory activity was demonstrated on both HIV-1 replicative enzymes (IC_50_ values ranged from 3.7–22 µM), while luteolin 7-*O*-glucoside selectively inhibited HIV-1 IN. In the case of betulinic acid, ursolic acid, and oleanolic acid, the inhibitory effect observed was selective for HIV-1 RT [113].

The aqueous and methanolic extracts of the pericarp of pomegranate were tested against HIV-1 PR, where they exhibited percentages of inhibition of 25.9 ± 4.2 and 18.0 ± 1.4, respectively [114]. However, the aqueous root bark of pomegranate showed 88% of inhibition on HIV-1 PR in a concentration of 250 µg/mL, which can be considered active (>70%) [115].

On the other hand, recent investigations established the relationship between antioxidant/phytochemical levels and immunomodulation in animals as well as in humans. Thus, the management of immune diseases such as HIV through targeting oxidative stress or boosting the endogenous levels of antioxidants could represent a benefit to general health and the immune system. In this respect, ellagic acid extracted from *P. granatum* as one of the major compounds has exhibited to be immunostimulatory in HIV [116].

Concerning the anticandidal properties of pomegranate, a number of investigations have demonstrated the effect of pomegranate on different strains of *Candida* spp., but mainly on *C*. *albicans* species. Among these investigations, as follows, we highlight some examples. The hydroalcoholic extract of the fruit peel of pomegranate exhibited high activity against *C. albicans* and *C. parapsilosis*, both exhibiting MICs of 3.9 µg/mL. By bio-guided fractionation, punicalagin was isolated as the majority compound and was tested as a pure compound against *C. albicans* and *C. parapsilosis* with the MICs obtained being 3.9 and 1.9 µg/mL, respectively. Moreover, the potent synergistic effect between punicalagin and Fluconazole against *C. albicans* was shown by the two-fold decrease in MIC values when they were combined. Combinations of punicalagin and other commercial drugs such as Amphotericin B, Nystatin, and Ketoconazole were also tested against *C. albicans*; however, synergism was observed only with Ketoconazole. In the same study, remarkable morphological alterations caused by punicalagin on *Candida* were observed by electron microscopy, including an irregular budding pattern and pseudohyphae, while certain ultrastructure alterations were also detected, such as a thickened cell wall, changes in the space between the plasma membrane and the cell wall, changes in vacuoles, and a reduction in cytoplasmic content [117].

Another investigation reported that the chitin-binding lectin (PgTeL) isolated from the fruit of pomegranate possesses anticandidal activity against *C. albicans* and *C. krusei*, due to that the observed values for the MIC were 25 and 12.5 µg/mL, respectively. The mechanisms involved in this anticandidal action comprise oxidative stress, energetic collapse, and damage to fungal cell walls. Indeed, PgTeL caused ultrastructural damage in both strains, but more prominent effects were observed in *C. krusei*, due to the alterations in the integrity of the fungal cell wall. Additionally, treatment of the yeast cells with PgTeL induced a decrease in intracellular ATP content and in lipid peroxidation. Even in lower concentrations (0.195 and 0.39 µg/mL), PgTeL exhibited significant antibiofilm activity on *C. albicans* [118].

An interesting report of the antimicrobial activity of the fruit peel ash extract of *P. granatum* was published in the year 2011. One half of the fresh fruit peels obtained in Turkey were combusted at 400 °C, and the other half was pulverized by using a mortar. Both were extracted by using a mix of solvents (dH_2_O:ethanol:methanol:acetone:CH_2_Cl_2_ (1:2.5:2.5:2:2)). The extract revealed anticandidal activity against *C. albicans* ATCC 26555 at the doses of 10, 20, and 30 µL [119].

The anticandidal activity of the extracts prepared with methanol, ethanol, water, acetone, chloroform, ethyl acetate, and methyl acetate of the fruit peels of *P. granatum* were evaluated in three *C. albicans* strains, *C. albicans* H from a human source, *C. albicans* V from chicken, and *C. albicans* N (NRRL YB-3464), by agar diffusion assay and broth microdilution susceptibility test. Although the extracts demonstrated anticandidal activity, the methanol, ethanol, and aqueous extracts exerted the best inhibitory effect on *C. albicans* growth. Additionally, the methanolic, ethanolic, and aqueous extracts were also employed in the preparation of an aerosol useful for completing the sanitization of semi-closed places against the growth of *C. albicans* [120].

In the year 2006, Vasconcelos et al. [121] evaluated the anticandidal properties of a gel prepared with the fruit of pomegranate against *C. albicans*, but also against the bacteria *S. mutans*, *S. mitis*, and *S. sanguis*. In the MIC adherence values of the pomegranate gel, *C. albicans* exhibited higher values than the bacteria. However, when *C. albicans* was associated with bacteria, the pomegranate gel exhibited stronger inhibition, particularly in the *S. mutans* + *S. mitis* + *S*. *sanguis* + *C. albicans* association. When compared with Miconazole, the pomegranate gel exhibited greater efficiency in inhibiting microbial adherence. Previously, these same authors reported an investigation of the in vivo evaluation of the gel prepared with the fruit of pomegranate against *C. albicans* associated with denture stomatitis. Thus, groups of 30 patients received the pomegranate gel 3 times per day for 15 days. The clinical results demonstrated a medium to good response in 21 subjects who received the pomegranate gel; in addition to that, a negativity for yeasts was observed in 23 subjects in whom pomegranate gel was administered. These results were very similar to those of the administration of the Miconazole gel, and perhaps the extract of pomegranate would be useful as a topical agent in the treatment of candidiasis associated with denture stomatitis [122].

A study on the evaluation against *Enterococcus faecalis* and *C. albicans* in isolation and in mono- and polymicrobial biofilms of the leaf hydroalcoholic extract of pomegranate, in combination with calcium hydroxide (Ca(OH)_2_) or alone, was carried out by Sousa et al. [123]. The persistent infections observed after endodontic procedures have rendered it possible to identify *E. faecalis* and *C. albicans* as the cause of these infections, in that they are frequently present in the root canal system. In order to disinfect the root canal, Ca(OH)_2_ has been widely used to delay dressing in the treatment of the canal systems. Therefore, novel substances that present antimicrobial actions against *E. faecalis* and *C. albicans*, and that additionally can be employed in combination with Ca(OH)_2_, represent an alternative of high value for endodontic treatments. The results showed that the pomegranate extract alone or in combination with Ca(OH)_2_ exhibited significant antimicrobial activity against planktonic cells and mono- and polymicrobial biofilms. Thus, pomegranate extract + Ca(OH)_2_ may be useful in endodontic treatments, due to the efficacy shown in disinfecting root canal systems.

The dichloromethane and methanol extracts of *P. granatum* were tested against several *Candida* strains, such as *C. albicans* CBS-562, *C. dubliniensis* CBS-7987, *C. parapsilosis* CBS-604, *C. tropicalis* CBS-94, *C. guilliermondii* CBS-566, *C. utilis* CBS-5609, *C. krusei* CBS-573, *C. lusitaniae* B-06, *C. glabrata* B-07, and *C. rugosa* B-12. While both extracts exhibited activity on all of the *Candida* species, the strongest effect was observed in the dichloromethane *P. granatum* extract. MIC values for both extracts ranged from 0.03–0.001 mg/mL [124].

The methanolic extracts of different organs of *P. granatum*, such as peel, flower, leaf, and stem, collected in Iran, were evaluated against *C. albicans* NCPF 3153 by well diffusion. The MIC and Minimum Fungicidal Concentration (MFC) values obtained revealed that the methanolic extract of leaf was the most active: 15.62 and 7.81 mg/mL, respectively. In the case of the maximal inhibition zones of the antifungal effect, the flower extract showed activity in the 200 μL concentration [125].

The aqueous and methanolic extracts of fruit skin of *P. granatum* were tested against *Pseudomonas aeruginosa*, *Staphylococcus aureus*, and *C. albicans* clinical isolates. The methanolic extract exhibited a potent antifungal effect on *C. albicans*, while both extracts exerted antibacterial activity [126]. The aqueous leaf and pericarp extracts of *P. granatum* were tested against the following *Candida* species: *C. albicans*, *C. glabrata*, *C. parapsilosis*, and *C. tropicalis*. The aqueous pericarp extract exerted an effect on *C. glabrata* and *C. albicans* with a zone of growth inhibition between 24 mm and 37 mm, respectively [127].

## 6. Discussion

Pomegranate has been employed extensively throughout centuries in the traditional medicine of ancient cultures for the treatment of a wide range of diseases, and at present, it has also been considered as functional food due the content of a large number of constituents that possess impressive roles in the management of health [128]. Although there is a high variety of phytochemicals of pomegranate present in the different organs of the tree, the polyphenols, and particularly, the anthocyanins and gallotannins, have been recognized as the main bioactive chemicals in pomegranate [20].

To date, pomegranate extracts have exhibited several properties, such as antioxidant, antibacterial, antifungal, anti-inflammatory, antiviral, and antidiabetic, among others; however, the antioxidant activities of pomegranate are among the most studied due to the potent effect exhibited by the extracts and isolated compounds, where punicalagin, ellagic acid, and anthocyanins have been recognized as the active phytochemicals mainly involved in the potent antioxidant properties of the pomegranate, and even more so, punicalagin has also been identified as causing more than one half of the pomegranate juice antioxidant effect [37,66].

Notwithstanding, in the case of other pharmaceutical properties of pomegranate, such as anti-HIV and anticandidal, studies remain scarce. However, in this present contribution, the literature reviewed revealed that the crude extracts of pomegranate demonstrated activity on different HIV targets, including the binding viral proteins (gp120) [110], and mainly, on the three enzymes involved in HIV replication, RT, PR, and IN [113,114]. The isolated compounds punicalagin and ellagic acid also exhibited inhibition in two replicative HIV enzymes: RT and IN [113]. The possibility of a single natural product, in this case, pomegranate, possessing two or more pharmacological effects, situates it in a predominant place. This fact acquires relevance in that any measure that decreases the pharmacological combinations will promote a better therapeutic result, mainly by improving adherence. Thus, the relevance of the pomegranate has also been considered due the crucial role that oral cavity health displays from the diagnosis of the disease until the adherence of the pharmacological treatment in patients with HIV/AIDS [11] (Table 3).

In addition, in the literature considered in this review, the potent anticandidal activity shown by the pomegranate clearly inhibited *C. albicans*, the species mainly considered in the diagnosis of HIV [117]. However, other species of *Candida* were also the target of pomegranate, such as *C. dubliniensis*, *C. guilliermondii*, *C. glabrata*, *C. krusei*, *C. lusitaniae*, *C. parapsilosis*, *C. rugosa*, *C. tropicalis*, and *C. utilis*. Therefore, pomegranate extracts and the majority compound, punicalagin, revealed anticandidal effects on a wide range of *Candida* strains, even clinically isolated strains. In particular, this anticandidal effect was exhibited in all of the extracts that were prepared with the fruit of the pomegranate, while extracts derived from other plant organs of pomegranate, such as flower, leaf, and stem, exhibited less potency [125]. In this manner, the potential use of pomegranate as an anti-HIV and anticandidal treatment in the oral cavity for the treatment of HIV appears to be a realizable alternative that additionally has the advantage of employing different organs of the plant and not only the fruit, with which the remainder of the plant’s organs, considered as industrial waste, would also be utilized.

To date, there is no cure for HIV/AIDS. Therefore, the search for novel drugs continues to be essential, and in particular, the search for drugs that exhibit multi-target action, in that the effectiveness of viral load control shown by the current pharmacological treatment for HIV/AIDS includes a combination of ARV with different enzymatic targets [12]. Thus, the literature reviewed in the present contribution permits us to appreciate the potential of the pomegranate as a source of bioactive compounds that would be useful in HIV/AIDS therapy due to its anti-HIV and anticandidal properties. Moreover, the traditional use of pomegranate in oral/dental diseases and its potent antioxidant properties also revealed that pomegranate would contribute to alleviate HIV/AIDS in a variety of ways. Indeed, according to the literature reviewed, pomegranate has been recommended clinically not only as a drug in the treatment of HIV/AIDS [110,111,113] but also as a food ingredient, due its inhibitory effects revealed in different steps of the HIV viral replication cycle, the rich content of bioflavonoids that contribute to the inhibition of free radicals, as well as to the inhibition of lipoxygenases [37].

Oral lesions, specifically chronic or recurrent fungal infections related to HIV infection, remain preponderant in the course and clinical progression of HIV infection. To date, and despite the enormous efforts and scientific contributions that have been made, they remain a critical problem in HIV-related morbidity [25]. Two issues have not been resolved in this regard: the toxicity of antifungals and the development of resistance by *Candida* species or the colonization of *Candida* species resistant to antifungals. Some antifungals have been reported to increase their power by acting by contact (topically) [27]. To our knowledge, no local side effects, such as type I hypersensitivity or contact stomatitis, have been reported. Therefore, in addition to their anti-HIV and anticandidal impact, it makes *P. granatum* extracts and punicalagin excellent candidates for studying their possible clinical application in the control of HIV viremia, and concomitantly, in the treatment of oral candidiasis related to AIDS. Due to that the pomegranate has been employed widely in traditional medicine for dental diseases without signs of toxicity, its use in the treatment of HIV/AIDS as antiviral and anticandidal treatment in the oral cavity would perhaps represent an alternative for HIV/AIDS patients. Moreover, the use of pomegranate in this treatment represents an alternative possessing other advantages: an easily available natural source that is inexpensive and safe [109].

Additionally, in the literature reviewed in the present contribution, the variety of pharmaceutical forms prepared with pomegranate, such as gel, mouthwash, mouthwash with silver nitrate nanoparticles, chewable tablets, silver nitrate nanoparticles, and aerosol [89,92,95,102,104,120], indicates that pomegranate can be manipulated without complications in order to elaborate the necessary pharmaceutical form according to the needs of patients. Regarding anti-oral candidiasis treatment, it still faces some challenges due to the humidity and surface tension of the oral mucosa that makes the adhesion to surfaces difficult. To date, some systems for slow or sustained release of oral antifungal agents have been proposed that could be effective in this respect. For example, the following nanoparticulate drug delivery systems and mucoadhesive drug delivery systems have been proposed as the most hopeful for successful oral treatment of OC: bioadhesive hydrogel to release Histatin-5; microemulsion of Clotrimazole-containing nanofibers; Amphotericin B cubosomal nanoparticles; fast-dissolving orodispersible films with Amphotericin B; microparticles of Itraconazole; nanosponges containing Voricozanol; mucoadhesive nanoparticles charged with Ketoconazole; Nystatin nanoemulsion; buccal film of Miconazole; self-emulsifying Voriconazole; Voriconazole sustained release tablet; mucoadhesive tablet of Miconazole; and Clotrimazole in situ gel [129,130]. These have allowed the evaluation of pomegranate in clinical assays. In this same regard, the literature reviewed divulged that pomegranate products have shown notorious synergistic effects with other commercial drugs such as antibiotics and antifungals [117], but also with substances that are useful in the dental area, such calcium hydroxide [123]. Regarding OC treatment and punicalagin, it has been reported that the combination of Nystatin and punicalagin increased antifungal efficacy and exhibited high efficacy in inhibiting *Candida* without showing cytotoxicity for keratinocytes [131]. Thus, the synergistic effects of pomegranate products represent a topic of study that still remains unexplored. More research protocols specifically focused on exploring the possible synergistic combination of *P. granatum* metabolites with other antifungal agents, where Amphotericin B could be a primary research target, are desirable. The synergistic effects among the active polyphenols of pomegranate and other components of the extract itself are attractive, since the multiple actions of complex mixtures that could be taking place in the crude extract cannot be ruled out, which is a constant in traditional herbal medicine [66,132]. Thus, the synergistic effects of pomegranate are encouraging future research due to the phytochemical content in pomegranate extracts, but also because of their interactions with other substances with different bioactivities.

Although the main objective of this review is to describe the anti-HIV and anticandidal effects of *P. granatum*, bearing in mind that OC is the most prevalent infectious oral disease in subjects living with HIV, it is evident that knowing the anticandidal properties of *P. granatum* is helpful for the treatment of OC not associated with HIV infection. In addition, the case of oral mucositis is noteworthy. Oral mucositis is one of the severe complications of chemotherapy and radiotherapy. Oral mucositis is characterized by erosion and ulceration of oral mucosa with erythema. Oral mucositis is significantly symptomatic, with pain and difficulty in swallowing, leading to nutritional deficiency and, of principal importance to the present paper, superimposed infections principally by *Candida* spp., the situation of which is even more dramatic in the case of the pediatric oncological patient. Mucositis increases morbidity and mortality in a large number of cancer patients. Restoring the integrity of the oral mucosa is fundamental in these patients. Unfortunately, to the best of our knowledge, there are no specific reports on the efficiency of *P. granatum* metabolites in treating oral mucositis. However, its usefulness in the repair of severe mucosal injury in the small bowel of rats treated with Methotrexate due to pomegranate juice accelerated intestinal mucosal repair and enhanced enterocyte turnover and could have clinical utility in cancer patients suffering chemotherapy-induced oral mucositis. Further studies are necessary to accept or refute the previous statement [133,134].

Taking into account the reviewed literature in the present contribution, pomegranate appears to be an excellent candidate to explore and incorporate in the treatment of the oral cavity in patients with HIV/AIDS, because, in addition to its antiviral and anticandidal pharmacological effects, the pomegranate represents an easily available natural source that is inexpensive and safe.

## Figures and Tables

**Table 1:** Main chemical content of *P. granatum*.

Part of the Plant	Chemical Group	Compound	Reference
Fruit (pericarp, peel, skin, rind)	Anthocyanidins	Cyanidin, delphinidin, pelargonidin	[43,44]
	Hydrobenzoic acids	Gallic acid, ellagic acid	[44,45,46]
	Hydroxycinnamic acids	Caffeic acid, chlorogenic acid, *p*-coumaric acid	[45]
	Alkaloids	Pelletierine, caffeine	[43,47]
	Tannins	Punicalagin, corilagin, casuarinin, punicallin, gallagyldilacton, pedunculagin, tellimagrandin, granatin A, granatin B, strictinin, punigluconin	[45,46]
	Flavonoids	Quercetin, rutin, kaempferol, luteolin, naringin, catechin, kaempferol-3-*O*-glycoside, kaempferol-3-*O*-rhamnoglucoside, hesperetin, hesperidine, isoquercetrin, naringin, naringenin	[43,46,47]
Juice	Anthocyanidins	Cyanidin, delphinidin, chrysanthemin, pelargonidin, cyanidin-3-*O*-glucoside, cyanidine-3,5-di-*O*-glucoside, delphinidin-3-*O*-glucoside, delphinidin-3,5-di-*O*-glucoside, pelargonidin-3-*O*-glucoside, pelargonidin-3,5-di-*O*-glucoside	[43,46,48]
	Hydrobenzoic acids	Quinic acid, callic acid, ellagic acid, protocatechuic acid	[46,49]
	Hydroxycinnamic acids	Caffeic acid, chlorogenic acid, *p*-coumaric acid	[46,49]
	Alkaloids	Pelletierine	[43]
	Tannins	Punicalagin, corilagin, casuarinin, punicallin, gallagyldilacton, punicalagin B, punicalagin D	[50,51]
	Flavonoids	Quercetin, rutin, naringin, kaempferol, luteolin, catechin, epicatechin, epigallocatechin-3-gallate, isoquercetin	[49,50,51]
Seeds	Fatty acids	Punicic acid, linoleic acid, oleic acid, palmitic acid, stearic acid, heneicosanoic acid, nonadecanoic acid, eicosenoic acid	[46,52]
	Hydrobenzoic acids	Ellagic acid, 3,3′-di-*O*-methylellagic acid, 3,3′,4′-tri-*O*-methylellagic acid	[52]
	Sterols	Stigmasterol, *β*-sitosterol, daucosterol, campesterol, cholesterol, 17-α-estradiol, estrone, testosterone, estriol	[52,53]
	Triterpenes	Ursolic acid, oleanolic acid, asiatic acid, betulinic acid	[46,52]
	Lignins	Coniferyl-9-*O*-[*β*-dapiofuranosyl (1→6)-*O*-*β*-D-glucopyranoside, sinapyl-9-*O*-[*β*-D-apiofuranosyl (1→6)-*O*-*β*-D-glucopyranoside, phenylethyl rutinoside, icariside D1	[54]
Flowers	Anthocyanidins	Pelargonidin 3,5-diglucoside, pelargonidin 3-glucoside	[35,55,56]
	Hydrobenzoic acids	Ellagic acid, gallic acid	[53,55]
	Triterpenes	Ursolic acid, oleanolic acid, maslinic acid, asiatic acid	[53,57]
	Flavonoids	Punicaflavone, tricetin, biochanin A	[55]
	Sterols	Estradiol	[57]
	Tannins	Punicalagin	[55]
Leaves	Anthocyanidins	Cyanidin	[52]
	Hydrobenzoic acids	Gallic acid, ellagic acid	[52]
	Flavonoids	Luteolin, apigenin, luteolin 4′-*O*-glucopyranoside, luteolin 3′-*O*-glucopyranoside, apigenin 4′-*O*-glucopyranoside	[53,57]
	Tannins	Punicalin, punicafolin, punicalagin, corilagin, granatin A, granatin B, 1,2,4-tri-*O*-galloyl-*β*-glucopyranose, 1,3,4-tri-*O*-galloyl-*β*-glucopyranose, 1,4-di-*O*-galloyl-3,6-(*R*)-hexahydroxydiphenyl-*β*-glucopyranose, 1,2,3-tri-*O*-galloyl-*β*-^4^C_1_,-glucose, brevifolin, tellimagrandin I	[58,59]
Roots	Alkaloids	Pelletierine, methylpelletierine, methyl-isopelletierine, isopelletierine, pseudopelletierine, sedridine, N-acetyl-sedridinehygrine, norhygrine, 2-(2′-hydroxypropyl) ∆1-piperideine, 2-(2-propenyl) ∆1-piperideine	[57,60,61,62]
	Tannins	Punicalin, punicalagin	[35,57]

**Table 3:** Pharmacological effects of the fruits of *P. granatum* in oral diseases.

Oral Disease	Part of the Fruit/ Pharmaceutic Preparation	Microbial/ Measure of the Effect	Effect	Reference
Dental caries	Juice and hydroalcoholic extract of peel	*Streptococcus* *mutans* ATCC^®^ 25175™ and *Rothia dentocariosa* clinical isolate	Peel extract inhibited *S*. *mutans* ATCC 25175 strain and *R*. *dentocariosa* clinical isolate with 10 μg/μL and 15 μg/μL of MIC and MBC values, respectively. Pomegranate juice exhibited high inhibitory activity against *S*. *mutans* ATCC 25175 strain with an MIC = 25 μg/μL and MBC value of 40 μg/μL, whereas against *R*. *dentocariosa*, showed MIC and MBC values of 20 μg/μL and 140 μg/μL, respectively.	[87]
	Two mouth rinses (pomegranate fresh juice and pomegranate peel extract)	*Streptococcus mutans* clinical isolate	Peel extract mouthwash exhibited potent reduction in *Streptococcus mutans* count (CFU) (100%), followed by fresh pomegranate juice (99.75%) in 45 children aged 5–10 years.	[88]
	Chewable tablets containing pomegranate ethanolic peel extract	*Streptococcus mutans* (MTCC 497t)	MIC value = 6.24 mg/mL. Formulating chewable tablet is better than mouth washes regarding stay-in-mouth time with good organoleptic properties.	[89]
	Aqueous pomegranate peel extract	Salivary pH	Aqueous pomegranate peel extract showed increase in the salivary pH (pH = 8.1) after 10 and 30 min of use in children aged between 6 and 8 years.	[90]
	Juice	*Streptococcus mutans* (MTCC) 890	The zones of inhibition of *S. mutans* were statistically highly significant (*p* = 0.008 and *p* = 0.007) in doses of 300 and 600 mg/mL.	[91]
Gingivitis	Mouthwash prepared with the entire fruit	Gingival index	Reduction in the gingival score (43.86%) after 15 days, better than the control, tested in 20 patients.	[92]
	Hydroalcoholic peel extract	Gingival index and IL-1β	Peel extract showed clinical and statistical significance for gingival index and the reduction in IL-1β levels due to the anti-inflammatory effects of peel extract.	[93]
	PomElla^®^ extract dissolved in water (standardized to 30% punicalagins; dose of 100 mg/day of flavonoids)	Total protein of saliva	↓ total protein ↓ activities of aspartate aminotransferase ↓ alpha-glucosidase activity ↑ activities of the antioxidant enzyme ceruloplasmin ↑ radical scavenging capacity	[94]
	Seed extract gel (10% concentration)	IL-1β, IL-8 and, CCL28 levels from Gingival Crevicular Fluid (GCF) in the first 14 days. After 14 and until 60 days: gingival index (GI), Bleeding on Probing (BOP), and Probing Depth (PD).	↓ IL-1β ↓ IL-8 = CCL28 ↓ GI ↓ BOP = PD	[95]
	Hydroalcoholic peel extract	Plaque Index (PI), Bleeding on Probing Index (BPI), oral streptococci from saliva samples	↓ PI ↓ BPI ↓ streptococci	[96]
Periodontitis	Juice	*Treponema denticola*, *Porphyromonas gingivalis*, and *Aggregatibacter actinomycetemcomitans*	Pomegranate juice caused a significant decrease in the biofilm mass of the three microbes but at different concentrations: *P. gingivalis* with 100% concentration; *A. actinomycetemcomitans*, 50% concentration; and *T. denticola*, 25% concentration.	[97]
	Mouthwash prepared with the fruit without peel	Plaque Index (PI), Bleeding Index (BI)	↓ PI ↓ BI	[98]
	Ethanolic peel extract	*Trichomonas tenax*	Potent anti *T. tenax* activity was recorded for pomegranate ethanol extract (60%).	[99]
	Glycolic extract of the whole fruit	*Porphyromonas gingivalis*	Glycolic extract exhibited in vivo antimicrobial activity against *P*. *gingivalis* using *Galleria mellonella* model.	[100]
Dental plaque	Hydroalcoholic extract of the entire fruit	*Staphylococcus aureus*, *S. epidermidis*, *S. β-hemolyticus*, *Pseudomonas* spp., *P. aeruginosa*, *Klebsiella pneumoniae*, *Proteus vulgaris*, *Escherichia coli*, and *Candida albicans*	Potent antimicrobial effect at the minimal dose tested (15 mg/mL). Microbials were isolated from 60 patients with good oral health (no caries, no gingivitis).	[101]
	Mouthwash prepared with the whole fruit	Plaque index	Reduction in plaque score (22.13%) after 15 days, tested in 20 patients.	[92]
	Mouthwash that contained silver nitrate nanoparticles using peel crude extract and its methanolic fraction	*Lysinibacillus* *cresolivorans*, *L. cresolivorans*, and *L. boronitolerans*	Inhibition zone ranging from 18.03–29.60 mm, exhibiting a potent effect. Cytotoxic activity against the normal skin fibroblast (BJ-1) showed LC_50_ values >134 µg/mL after 48 hr, showing that the pomegranate extract is safe.	[102]
	Juice without sugar	Streptococci and Lactobacilli	Reduction in the number of colony forming units (CFU) was 23% for the case of Streptococci strains, while 46% was observed to Lactobacilli strains.	[103]
Oral cancer	Juice, punicalagin, ellagic acid, and a standardized total pomegranate tannin (TPT) extract	Antiproliferative (on KB, CAL27 human oral tumor cells) and antioxidant activities ((inhibition of lipid peroxidation and Trolox equivalent antioxidant capacity (TEAC))	Juice showed the greatest antiproliferative activity against cell lines by inhibiting proliferation from 30–100%. The trend in antioxidant activity was juice > TPT > punicalagin > ellagic acid.	[55]
	POMx, a commercial pomegranate-derived polyphenol-rich aqueous extract powder (POM Wonderful, LLC, Los Angeles, CA, USA)	Hep-2 cell proliferation, vascular endothelial growth factor (VEGF) expression, and caspase-3 expression	↓ cell proliferation ↓ VEGF expression ↑ caspase-3 expression	[104]
	POMx, a commercial pomegranate derived polyphenols-rich aqueous extract powder (POM Wonderful, LLC, Los Angeles, CA, USA)	Cytotoxicity (carcinoma cells lines: HSC-2, CAL27, and SCC1483) and apoptotic activities	Midpoint cytotoxicity (NR_50_) values were 100 g/mL for the HSC-2 and CAL 27 cells, and 125 g/mL for the SCC1483 cells, through activation of caspase-3, cleavage of poly(ADP-ribose) polymerase (PARP), and reduction in glutathione (GSH) levels	[105]
	Extract of the whole fruit	Expression of BCL-2 (proto-oncogene)	↓ BCL-2 expression in the malignant epithelial cells of the oral mucosa of mice	[106]
	Molasses (PM)	Transforming growth factor β (TGF-β) and Cytokeratin-19 (CK-19) expressions and dysplastic changes	↓ TGF-β ↓ CK-19 ↓ dysplastic changes	[107]

## Data Availability

Not applicable.

## Abbreviations

HIV	Human Immunodeficiency Virus
AIDS	Acquired Immunodeficiency Syndrome
HAART	Highly Active AntiRetroviral Therapy
OC	Oral Candidiasis
ARV	Antiretroviral
RT	Reverse Transcriptase
IN	Integrase
PR	Protease
PLWA	People Living With AIDS
ATCC	American Type Culture Colection
MRSA	Methicillin-Resistant Staphylococcus aureus
DSMZ	Leibniz Institute DSMZ
GmbH	German Collection of Microorganisms and Cell Cultures
NF-kB	Core Factor Kappa Beta
GCF	Gingival Crevicular Fluid
PI	Plaque Index
BPI	Bleeding on Probing Index
LC50	Letal Concentration 50
CFU	Colony Forming Units
MTCC	Microbial Type Culture Collection
TPT	Total Pomegranate Tannin
TEAC	Trolox Equivalent Antioxidant Capacity
VEGF	Vascular Endothelial Growth Factor
PARP	Poly(ADP-Ribose) Polymerase
GSH	Glutathione
TGF-β	Transforming Growth Factor Beta
CK-19	Cytokeratin-19
PgTeL	Chitin-Binding Lectin
MICs	Minimum Inhibitory Concentrations
MFC	Minimum Fungicidal Concentration
NCPF	National Collection of Pathogenic Fungi
CBS	Fungal Biodiversity Centre
